# Complement activation in the Parkinson's disease substantia nigra: an immunocytochemical study

**DOI:** 10.1186/1742-2094-3-29

**Published:** 2006-10-19

**Authors:** David A Loeffler, Dianne M Camp, Stephanie B Conant

**Affiliations:** 1 Division of Neurology, William Beaumont Hospital Research Institute, Royal Oak, MI 48073, USA; 2 Department of Microbiology and Immunology, Vanderbilt University School of Medicine, Nashville, TN 37232, USA

## Abstract

**Background:**

Inflammatory processes are increased in the Parkinson's disease (PD) brain. The long-term use of nonsteroidal anti-inflammatory drugs has been associated, in retrospective studies, with decreased risk for PD, suggesting that inflammation may contribute to development of this disorder. The objective of this study was to determine the extent of complement activation, a major inflammatory mechanism, in PD.

**Methods:**

Substantia nigra specimens from young normal subjects (n = 11–13), aged normal subjects (n = 24–28), and subjects with PD (n = 19–20), Alzheimer's disease (AD; n = 12–13), and dementia with Lewy bodies (DLB; n = 9) were stained for iC3b and C9, representing early- and late-stage complement activation, respectively. Numbers of iC3b^+^, C9^+^, and total melanized neurons in each section were counted in a blinded fashion. Nonparametric analyses were used to evaluate differences between groups and to evaluate correlations between complement staining, numbers of melanized neurons, and the duration of PD.

**Results:**

Lewy bodies in both PD and DLB specimens stained for iC3b and C9. Staining was also prominent on melanized neurons. The percentage of iC3b^+ ^neurons was significantly increased in PD vs. aged normal and AD specimens, and in young normal vs. aged normal specimens. C9 immunoreactivity was significantly increased in PD vs. AD specimens, but unlike iC3b, the increased C9 staining in PD and young normal specimens did not achieve statistical significance vs. aged normal specimens. iC3b and C9 staining in PD specimens was not correlated with the numbers of remaining melanized neurons, nor with the duration of PD.

**Conclusion:**

Complement activation occurs on Lewy bodies and melanized neurons in the PD substantia nigra. Early complement activation (iC3b) is increased on melanized neurons in PD vs. aged normal specimens, and late-stage complement activation (C9) also tends to increase. This latter finding suggests that complement activation may contribute to loss of dopaminergic neurons in some individuals with PD. Complement activation on melanized neurons appears to decrease with normal aging, suggesting a possible neuroprotective role for this process in the normal substantia nigra.

## Background

Multiple neurotoxic processes have been described in the Parkinson's disease (PD) brain including inflammation, oxidative stress, excitotoxicity, and mitochondrial dysfunction [1]. The evidence for inflammation in PD includes gliosis [2,3], increased major histocompatibility complex expression on microglia [2,4], microglial phagocytosis of degenerating neuromelanin-containing neurons [5], and increased inflammatory cytokines [6,7]. Inflammation has also been reported in some animal models of PD [8,9]. The significance of inflammation in PD is unclear. Two retrospective studies indicated an association between the long-term use of nonsteroidal anti-inflammatory drugs (NSAIDs) and decreased risk for PD [10,11], suggesting that inflammation may be important in the development of this disorder; however, a third retrospective study found no evidence for protective effects of NSAIDs against PD [12].

Complement activation is a major inflammatory process which promotes the removal of microorganisms and cell debris, and the processing of immune complexes. Three interrelated pathways, the classical, alternative, and mannan binding lectin-mediated cascades, have been described. Proteins generated early in this process function as chemotactic factors [13,14], opsonins [15,16], and anaphylatoxins [17]. Full activation of any of these pathways results in the generation of C5b-9, the membrane attack complex (MAC), which is neurotoxic [18]. In contrast to Alzheimer's disease (AD), in which complement activation has been extensively investigated [reviewed by McGeer and McGeer [19], 2002, and Shen and Meri [20], 2003], few studies have addressed this issue in PD. Yamada et al. [21] reported staining of Lewy bodies in the PD substantia nigra for both early-stage (C3d and C4d) and late-stage (C7 and C9) complement proteins, and C3d and C4d staining on Lewy bodies was subsequently reported in the brain stem from subjects with dementia with Lewy bodies (DLB) [22]. However, a third study found no complement reactivity on Lewy bodies in the cingulate gyrus in either PD or DLB [23]. Because of these conflicting results, the extent of complement activation in PD is unclear. The objective of the present study was to further examine this issue.

## Methods

### Brain specimens

Paraffin-embedded, formalin-fixed substantia nigra specimens were obtained from young normal (YN) subjects (n = 11–13), aged normal (AN) subjects (n = 24–28), and subjects with PD (n = 19–20), AD (n = 12–13), and DLB (n = 9). These specimens were obtained from the Harvard Brain Tissue Resource Center (McLean Hospital, Belmont, MA), the University of California at Irvine Institute for Brain Aging and Dementia (Irvine, CA), the Massachusetts General Hospital Alzheimer Disease Research Center (Charlestown, MA), and the University of California School of Medicine (Department of Medical Pathology, Sacramento, CA). Each group (YN, AN, PD, AD, and DLB) included specimens from all four brain banks. Means (± SEM) and ranges for subject ages and post-mortem intervals (PMI) are shown in Table 1. PMI means were similar between groups, and subject ages differed only between YN and the other groups.

**Table 1:** Subject ages and post-mortem intervals.

Group	n	Age (yrs)	Age range	PMI (hrs)	PMI range (hrs)
YN	11–13	43.2 ± 1.9^a^	24–53	15.8 ± 2.0	3.0–24.0
AN	24–28	83.7 ± 2.1	66–104	10.8 ± 1.4	0.3–23.0
PD	19–20	80.2 ± 2.1	66–91	11.2 ± 2.3	2.0–29.0
AD	12–13	76.8 ± 1.9	61–83	7.4 ± 1.6	3.0–23.3
DLB	9	78.3 ± 1.9	70–86	8.9 ± 2.0	1.6–16.3

PMI means were similar between groups, and subject ages differed only between young normal specimens and the other groups. Data are expressed as means ± SEM. (^a^*p *< 0.05 vs. other groups; abbreviations: AD, Alzheimer's disease; AN, aged normal; DLB, dementia with Lewy bodies; PD, Parkinson's disease; PMI, post-mortem interval; YN, young normal)

### Immunocytochemical staining for iC3b and C9

Formalin-fixed, paraffin-embedded sections of 6 – 8 μm thickness were placed on Superfrost Plus slides (Cardinal Health, McGaw Park, IL) and heated for 1 hr at 56°C. The sections were subsequently deparaffinized and rehydrated through graded ethanol baths, then rinsed in Tris buffered saline (TBS; 0.1 M Tris, 0.85% NaCl, pH 7.6). (This and all subsequent rinses were performed three times at five min intervals.) They were treated for 4 min with 88% formic acid (Fisher Scientific, Fair Lawn, NJ), then boiled for 5 min in citrate buffer, pH 6.0 (Antigen Unmasking Solution, Vector Laboratories, Burlingame, CA). After rinsing in TBS, the sections were treated with 3% H_2_O_2_/10% methanol in TBS for 30 min to eliminate endogenous peroxidase activity, rinsed in TBS with 0.1% Triton X-100 (hereafter, TBS-T), then treated with TBS-T with 1% bovine serum albumin (TBS-T-BSA) and 10% normal horse serum (Vector) for 30 min. The specimens were then incubated overnight at room temperature with mouse monoclonal anti-human iC3b (Quidel Corp., San Diego, CA; 1:200 dilution, final concentration 5.5 μg/ml) or goat anti-human C9 (Quidel; 1:5000 dilution, final concentration 11 μg/ml). Negative controls, performed for each specimen, consisted of substituting the nonsecreting mouse hybridoma MOPC-21 (mouse IgG1-kappa; Sigma-Aldrich, St. Louis, MO) (1:164 dilution, final concentration 5.5 μg/ml) for anti-iC3b serum, and normal goat serum (Vector; 1:5000 dilution) for goat anti-C9. After rinsing in TBS-T, biotinylated horse anti-mouse IgG (for iC3b staining) or biotinylated horse anti-goat IgG (for C9 staining) (both from Vector; 1:200 dilution in TBS-T-BSA) was applied at room temperature for one hr (for iC3b) or 90 min (for C9), followed by rinsing in TBS and then avidin-biotin-horseradish peroxidase conjugate (ABC reagent, Vector; 1:100 dilution in TBS-BSA) for 1 hr. Sections were developed with 3,3'-diaminobenzidine (DAB)/H_2_O_2 _with nickel enhancement (DAB Peroxidase Substrate Kit, Vector), then dehydrated in ethanol baths to xylene and coverslipped with Cytoseal-60 Mounting Medium (Richard-Allan Scientific, Kalamazoo, MI). AD hippocampus specimens from the University of California at Irvine Institute for Brain Aging and Dementia were included as positive controls in each experiment.

### Statistical analyses

The number of neuromelanin-containing neurons (hereafter, "melanized neurons") in each substantia nigra section (one side only), and the number of these neurons immunoreactive for iC3b or C9, were counted by one observer (D.L.) in a blinded fashion with the 40× objective. (Neuromelanin, a by-product of dopamine metabolism [24], is considered to be a marker for dopaminergic neurons in the substantia nigra, although some dopamine-containing neurons in this region are non-melanized [25]).

The percentage of iC3b^+ ^or C9^+^melanized neurons and number of melanized neurons in each specimen were compared between groups via the Kruskal-Wallis test and subject ages and PMI were compared between groups by a one-way ANOVA. When significant differences between groups were detected, pairwise comparisons were then performed to determine the location(s) of these differences. Data from iC3b^+^, C9^+^, and total melanized neuron counts were analyzed with a Wilcoxon Rank Sum test, with the *p*-values adjusted for multiplicity of testing via Hochberg's procedure [26]. The two demographic factors, subject age and PMI, were compared between groups in a pair-wise fashion via the Tukey-Kramer HSD. Correlations between variables (percentages of C3b^+ ^and C9^+^melanized neurons, PMI, age, number of melanized neurons, and duration of PD) were determined by Spearman's rank correlation coefficient. The overall level of statistical significance for all tests was 0.05.

## Results

**Figure 1:**
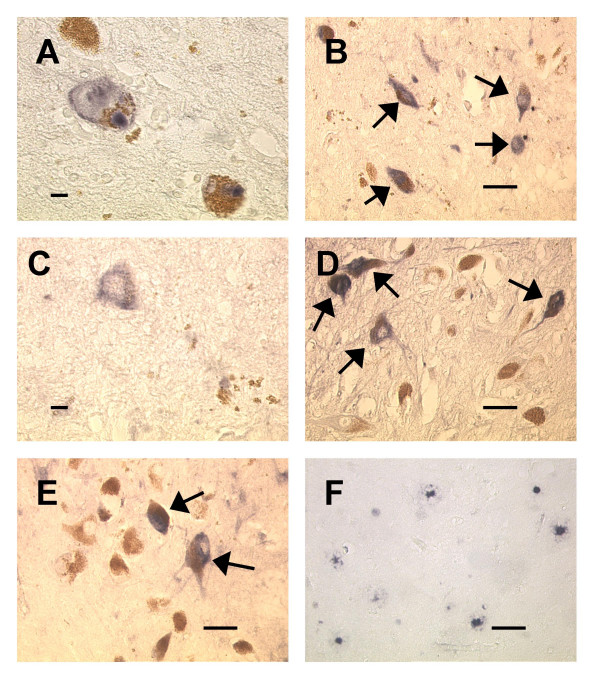
**iC3b staining in substantia nigra specimens**. Fig. 1A: Immunoreactive Lewy bodies in a PD substantia nigra specimen; Fig. 1B: Staining of melanized neurons (arrows) in a different PD specimen; Fig. 1C: Immunoreactive neuron with little melanin remaining, same PD specimen as Fig. 1B; Fig. 1D: iC3b staining of melanized neurons (arrows) in a young normal specimen; compare with unstained neurons in lower part of field; Fig. 1E: similar staining pattern in an AD specimen; two prominently stained melanized neurons are seen (arrows) among several unstained neurons; Fig. 1F: iC3b-stained senile plaques in a different AD substantia nigra specimen. (Figs. 1A and 1C, bar = 10 μm; Figs. 1B and 1D–F, bar = 50 μm; immunoreactive structures are dark blue or gray, in contrast to brown melanin and yellow background).

**Figure 2:**
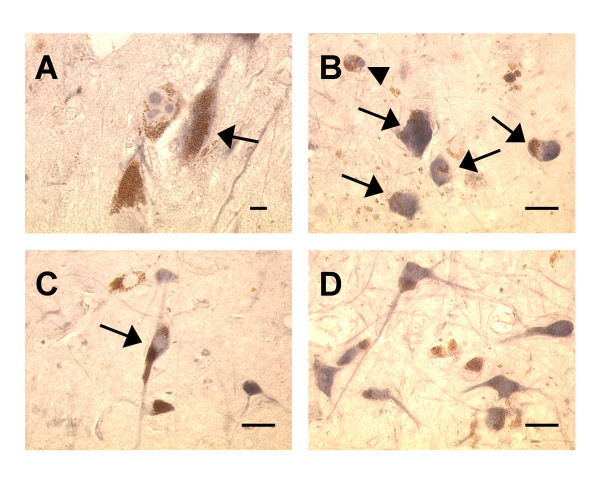
**C9 staining in substantia nigra specimens**. Fig. 2A: Staining of multiple Lewy bodies within a melanized neuron in a PD specimen; adjacent melanized neuron (arrow) and its axon are also C9-positive; Fig. 2B: immunoreactivity for C9 in a Lewy body (arrowhead) and in melanin-depleted neurons (arrows) in a different PD specimen; Fig. 2C: staining of melanized neuron (arrow) and its processes in a DLB specimen; Fig 2D: multiple immunoreactive melanized neurons in an aged normal specimen. (Fig. 2A, bar = 10 μm; Figs. 2B–D, bar = 50 μm; immunoreactive structures are dark blue or gray, in contrast to brown melanin and yellow background).

Lewy bodies were immunoreactive for both iC3b (7 of 20 PD specimens, 6 of 9 DLB specimens) and C9 (11 of 19 PD specimens, 9 of 9 DLB specimens). Staining was also detected on melanized neurons (cell bodies, axons, and melanin fragments), occasional non-melanized neurons, glia, and, in AD specimens, senile plaques. In PD specimens, many of the iC3b^+ ^and C9^+ ^melanized neurons had few remaining melanin granules. No cellular staining was present in negative controls, although faint vascular staining was observed in a few specimens. Staining for iC3b and C9 is shown in Figs. 1 and 2, respectively. There was marked variation in the percentages of immunoreactive melanized neurons for different specimens within each group, with little or no staining in some specimens and more than 25% staining in others; staining even exceeded 50% of melanized neurons in a few specimens. Complement immunoreactivity of melanized neurons generally was not localized to a particular sector (lateral, middle, or medial) of the substantia nigra.

**Figure 3:**
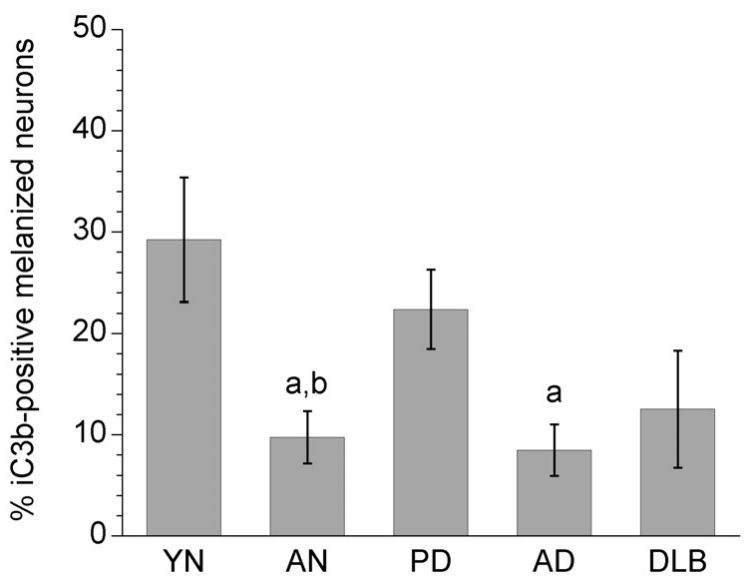
**Percentages of iC3b-positive melanized neurons in different groups of substantia nigra specimens**. The percentage of iC3b^+ ^melanized neurons was significantly increased in PD vs. both aged normal and AD specimens, and in young normal vs. aged normal specimens. Data are expressed as means ± SEM. (^a^*p *< 0.05 vs. PD; ^b^*p *< 0.05 vs. young normal specimens; abbreviations: AD, Alzheimer's disease; AN, aged normal; DLB, dementia with Lewy bodies; PD, Parkinson's disease; YN, young normal)

**Figure 4:**
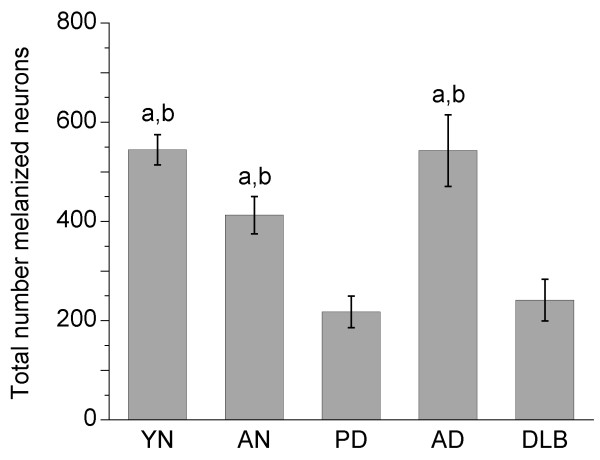
**Numbers of melanized neurons in different groups of substantia nigra specimens**. Total numbers of melanized neurons were significantly decreased in PD vs. aged normal, young normal, and AD specimens, and in DLB vs. aged normal and AD specimens. Data (means ± SEM) are shown for slides from specimens in which iC3b immunoreactivity was assessed; essentially similar results were obtained for slides from specimens in which C9 staining was evaluated. (^a^*p *< 0.05 vs. PD; ^b^*p *< 0.05 vs. DLB; abbreviations: AD, Alzheimer's disease; AN, aged normal; DLB, dementia with Lewy bodies; PD, Parkinson's disease; YN, young normal)

Statistical analysis of iC3b staining revealed significant differences among groups (*p *= 0.003), and pairwise comparisons indicated that the percentage of iC3b^+ ^melanized neurons was significantly increased in PD vs. both AN and AD specimens (*p *= 0.0011 and 0.0099, respectively), and in YN vs. AN specimens (*p *= 0.0146) (Fig. 3). Total numbers of melanized neurons were significantly decreased in PD vs. AN, YN, and AD specimens, and in DLB vs. AN and AD specimens (Fig. 4). iC3b immunoreactivity was significantly correlated with numbers of melanized neurons only in YN specimens (r = 0.63, *p *= 0.016). There was no correlation in PD specimens between the percentage of iC3b^+ ^melanized neurons and the duration of PD (r = 0.09), and no gender differences were detected on pooled data from all groups for iC3b staining.

**Figure 5:**
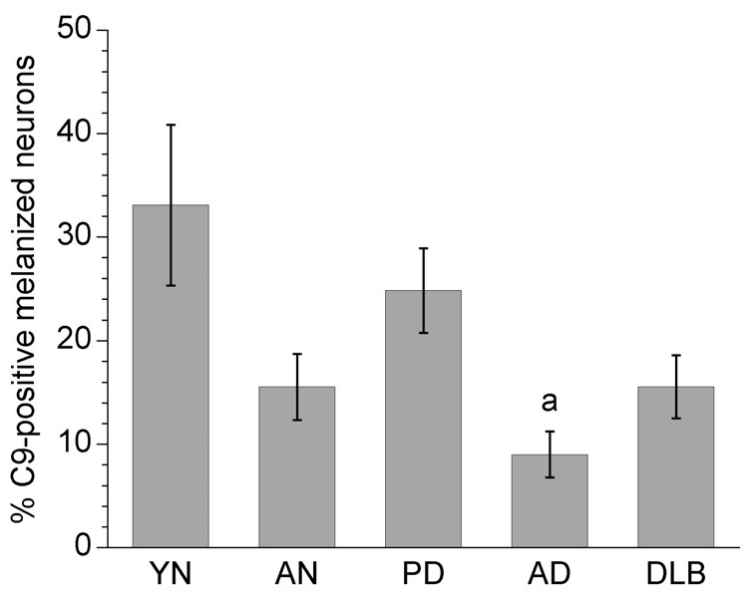
**Percentages of C9-positive melanized neurons in different groups of substantia nigra specimens**. C9 staining was significantly increased in PD vs. AD specimens. The percentages of C9^+ ^melanized neurons in PD and young normal specimens tended to be increased vs. aged normal specimens, but these differences were not significant (*p *= 0.04 [not significant after adjustment for multiple comparisons] and 0.08, respectively). Data are expressed as means ± SEM. (^a^*p *< 0.05 vs. PD; abbreviations: AD, Alzheimer's disease; AN, aged normal; DLB, dementia with Lewy bodies; PD, Parkinson's disease; YN, young normal)

C9 staining yielded generally similar results to those for iC3b. This was reflected by significant correlations between the percentages of C9^+ ^and iC3b^+ ^melanized neurons in all groups (r values ranging from 0.67 to 0.82, all *p *< 0.02) except for DLB (r = 0.35, *p *= 0.40). C9 staining was increased in PD vs. AD specimens (*p *= 0.0048; Fig. 5). Unlike iC3b, however, the trends towards increased C9 staining in PD vs. AN specimens, and in YN vs. AN specimens, were not statistically significant (*p *= 0.04 [not significant after adjustment for multiple comparisons] and *p *= 0.08, respectively). The percentage of C9^+ ^melanized neurons was not correlated with the number of melanized neurons per specimen in any of the groups. As with iC3b, neuronal C9 staining was not correlated with the duration of PD, and there were no gender differences within groups for C9 staining.

## Discussion

This study confirmed the presence of both early- and late-stage complement proteins on Lewy bodies in the PD substantia nigra, as reported by Yamada et al. [21]. In contrast to that study, however, complement activation was also detected on melanized neuron cell bodies and axons. These differences may be due to technical factors; the present study used on-slide staining of formalin-fixed sections and included antigen retrieval pretreatment of sections with formic acid and citric acid, whereas the earlier study used free-floating staining, primarily of paraformaldehyde-fixed sections, without antigen retrieval.

The antibody used to detect iC3b in this study is iC3b-specific and does not recognize the native complement protein C3 from which iC3b is generated. iC3b staining of melanized neurons is therefore evidence for early complement activation, i.e., cleavage of C3, on these cells. iC3b and its active form, C3b, are opsonins, promoting phagocytosis of foreign antigens and cell debris. Deposition of iC3b on melanized neurons could facilitate binding of these cells by activated microglia, known to be present in increased numbers in the PD substantia nigra [3]. C3a, the other major C3 cleavage protein, is an anaphylatoxin, increasing vascular permeability. Though C3a is generally considered to be pro-inflammatory [27-29] because it attracts and activates eosinophils, basophils, and mast cells, few of these cells are present in the brain. C3a may, in fact, limit brain inflammation, by decreasing the production of inflammatory cytokines and inducing the production of immunosuppressive ones [30]. It exerts neuroprotective and (indirectly) neurotrophic effects, protecting neurons against excitotoxins [31] and inducing production of microglial neuronal growth factor (NGF) [32]. iC3b staining of melanized neurons was greater in YN than in AN specimens, and was positively correlated with the numbers of melanized neurons in YN specimens (r = 0.63, *p *= 0.016). These results suggest that early complement activation might play a protective role for melanized neurons in the young normal brain; if so, a decrease in early complement activation on melanized neurons during normal aging could leave these cells more susceptible to oxidative and/or inflammatory damage. The decrease in iC3b staining of melanized neurons which occurred with normal aging was not detected when PD was present. The significance of this finding is unclear. The lack of correlation in PD specimens between the numbers of remaining melanized neurons and the percentage of these neurons that were iC3b^+ ^suggests that, even if early complement activation is primarily neuroprotective, this process fails to protect melanized neurons from whatever insults cause them to be lost in the PD brain.

Goat anti-C9 was used rather than monoclonal anti-C5b-9 for assessment of late-stage complement activation because, in preliminary studies, more consistent staining of senile plaques in AD hippocampus sections was obtained with the anti-C9 antibody. (AD brain was the appropriate positive control for these studies because extensive deposition of C5b-9 has been reported in the AD brain [33]). Although staining for C9 was also used in the study by Yamada et al. [21] and has been used by others to detect the MAC [34-36], C9 immunoreactivity on melanized neurons could indicate late-stage complement activation, upregulation of neuronal C9 synthesis, or both. C9 staining on melanized neurons tended to increase in PD vs. AN specimens (60% increase), although this increase was not statistically significant. Detection of C9 on degenerating melanized neurons suggests that deposition of the MAC on dopamine neurons may reach lytic levels in PD and contribute to the loss of these neurons. The mechanism by which complement is activated on PD melanized neurons is unknown; one possibility may be surface immunoglobulin G (IgG), which was recently reported by Orr et al. [37] to be present on 30% of dopamine neurons in the PD substantia nigra. Alternatively, complement activation on melanized neurons could occur secondary to cell injury, triggered by newly exposed tissue antigens and/or byproducts of damaged tissue, although this would not explain the apparent activation of complement on melanized neurons in the YN substantia nigra specimens.

The increase in iC3b immunoreactivity on melanized neurons in YN substantia nigra specimens in comparison with AN specimens was an unexpected finding. A similar trend was present for C9, although it was not statistically significant. The mechanism responsible for complement activation on normal dopamine neurons, as with injured dopamine neurons, is unknown. Oxidative stress, which can activate complement [38], may be involved. The basal level of oxidative stress in the human substantia nigra is higher than in other brain regions [39], probably due to the production of H_2_O_2 _as a byproduct of dopamine metabolism [40]. Early complement activation on normal dopamine neurons could play a protective role, as discussed earlier, whereas MAC deposition on these neurons, if it occurs, is likely to be sublytic. There is a substantial literature on the cellular effects of sublytic levels of the MAC, including cell cycle activation, cell proliferation, enhancement of cell survival, and cytokine synthesis [41-44], but its influence on neurons has apparently not been examined. In addition to the concentrations of complement proteins deposited on melanized neurons, neuronal expression of complement inhibitory molecules [45] and complement receptors [46] in normal and diseased substantia nigra is also likely to be important in determining the influence of complement activation on these neurons.

## Conclusion

This study confirms the occurrence of complement activation on Lewy bodies in melanized neurons in the PD substantia nigra, and indicates that this process also occurs on some non-Lewy body-bearing melanized neurons and on melanin fragments in this region. Complement activation on melanized neurons tends to increase in the PD substantia nigra, but is also present in normal individuals and in subjects with other neurodegenerative disorders. Complement activation on melanized neurons may decrease during normal aging. Further studies are indicated to clarify the mechanism (or mechanisms) responsible for complement activation on normal and injured dopamine neurons, and the significance of this process.

## Competing interests

The author(s) declare that they have no competing interests.

## Authors' contributions

DAL performed the immunocytochemical staining and cell counts and wrote the manuscript. DMC generated the figures, performed the statistical analyses, and assisted with the writing of the manuscript. SBC performed preliminary experiments to develop the staining methods and reviewed the manuscript.
